# {2,2′-[Ethane-1,2-diylbis(nitrilo­methan­yl­yl­idene)]diphenolato}(iso­propano­lato)aluminium di­chloro­methane hemisolvate

**DOI:** 10.1107/S1600536813029644

**Published:** 2013-11-06

**Authors:** Kirill V. Zaitsev, Ekaterina A. Kuchuk, Sergey S. Karlov, Galina S. Zaitseva, Andrei V. Churakov

**Affiliations:** a Department of Chemistry, M.V. Lomonosov Moscow State University, Leninskie Gory 1/3, Moscow 119991, Russian Federation; b Institute of General and Inorganic Chemistry, Russian Academy of Sciences, Leninskii prosp. 31, Moscow 119991, Russian Federation

## Abstract

In the title compound, [Al(C_16_H_14_N_2_O_2_)(C_3_H_7_O)]·0.5CH_2_Cl_2_, the salen complex is monomeric and the dichlormethane solvent mol­ecule lies on a crystallographic twofold axis. The central Al atom is fivefold coordinated and possesses a square-based pyramidal environment. The Al—OAlk(^*i*^prop­yl) bond [1.7404 (14) Å] is much shorter than the Al—OAr(salen) bond lengths [1.7974 (15) and 1.8094 (14) Å]. The iso­propyl­oxo group forms an intra­molecular C—H⋯N hydrogen bond. In the crystal, the complex mol­ecules are linked by weak C—H⋯O inter­actions.

## Related literature

For general background to the chemistry affording aluminium complexes based on salen-type ligands, see: Matsumoto *et al.* (2007 ▶); Gurian *et al.* (1991 ▶); Atwood *et al.* (1997 ▶); Muñoz-Hernandez *et al.* (2000 ▶). For our previous work on main group element complexes with polydentate *N*,*O*-ligands, see: Karlov & Zaitseva (2001 ▶). For structures of related monomeric Al-salen complexes, see: Darensburg & Billodeaux (2005 ▶); Gurian *et al.* (1991 ▶); Pang *et al.* (2008 ▶). For a description of the Cambridge Structural Database, see: Allen (2002 ▶).
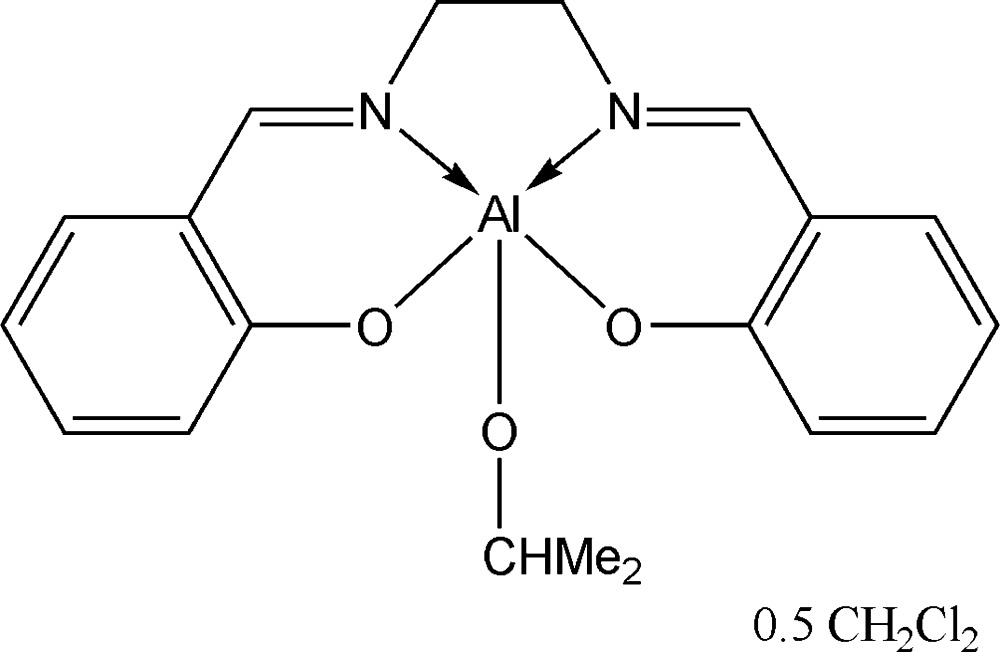


## Experimental

### 

#### Crystal data

[Al(C_16_H_14_N_2_O_2_)(C_3_H_7_O)]·0.5CH_2_Cl_2_
*M* *_r_* = 394.82
Orthorhombic, 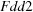

*a* = 24.427 (3) Å
*b* = 30.875 (4) Å
*c* = 10.0956 (13) Å
*V* = 7614.0 (16) Å^3^
*Z* = 16
Mo *K*α radiation
μ = 0.27 mm^−1^
*T* = 173 K
0.25 × 0.15 × 0.04 mm

#### Data collection

Bruker SMART APEXII diffractometer
Absorption correction: multi-scan (*SADABS*; Bruker, 2008 ▶) *T*_min_ = 0.936, *T*_max_ = 0.989
13794 measured reflections
4069 independent reflections
3687 reflections with *I* > 2σ(*I*)
*R*_int_ = 0.032

#### Refinement

*R*[*F* ^2^ > 2σ(*F* ^2^)] = 0.034
*wR*(*F* ^2^) = 0.081
*S* = 1.04
4069 reflections
242 parameters
1 restraint
H-atom parameters constrained
Δρ_max_ = 0.32 e Å^−3^
Δρ_min_ = −0.32 e Å^−3^
Absolute structure: Flack (1983 ▶), 1872 Friedel pairs
Absolute structure parameter: −0.09 (7)

### 

Data collection: *APEX2* (Bruker, 2008 ▶); cell refinement: *SAINT* (Bruker, 2008 ▶); data reduction: *SAINT*; program(s) used to solve structure: *SHELXTL* (Sheldrick, 2008 ▶); program(s) used to refine structure: *SHELXTL*; molecular graphics: *SHELXTL*; software used to prepare material for publication: *SHELXTL*.

## Supplementary Material

Crystal structure: contains datablock(s) I. DOI: 10.1107/S1600536813029644/fk2075sup1.cif

Structure factors: contains datablock(s) I. DOI: 10.1107/S1600536813029644/fk2075Isup2.hkl

Supplementary material file. DOI: 10.1107/S1600536813029644/fk2075Isup3.mol

969040

Additional supplementary materials:  crystallographic information; 3D view; checkCIF report

## Figures and Tables

**Table 1:** Hydrogen-bond geometry (Å, °)

*D*—H⋯*A*	*D*—H	H⋯*A*	*D*⋯*A*	*D*—H⋯*A*
C1—H1*A*⋯N1	1.00	2.64	3.204 (3)	116
C18—H18*A*⋯O3^i^	0.99	2.55	3.508 (2)	163
C4—H4⋯O1^ii^	0.96	2.33	3.2539 (18)	160

Symmetry codes: (i) 
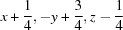
; (ii) 
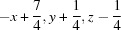
.

**Fig. 1.**
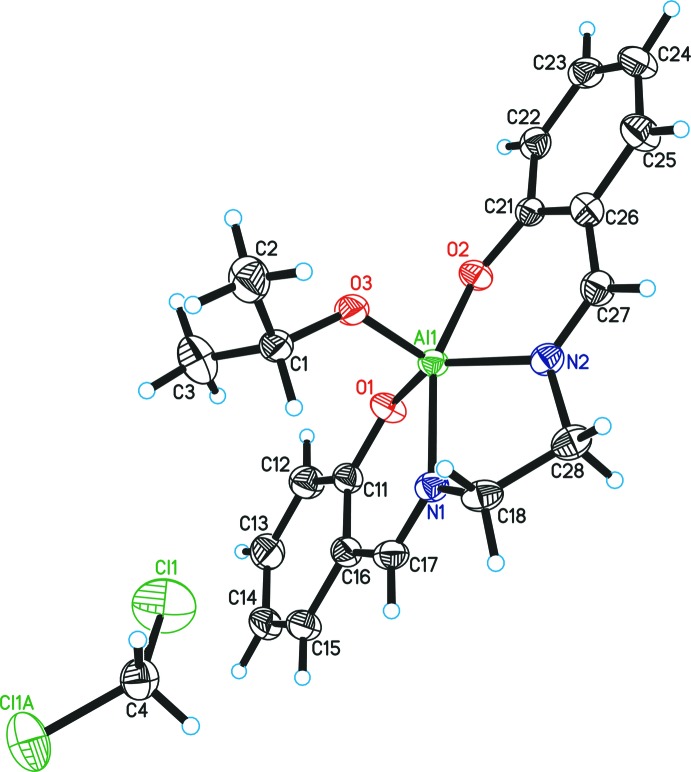
The molecular structure of the title compound. Anisotropic displacement ellipsoids are shown at the 50% probability level. [Symmetry code: (A) 2-x, 1-y, z]

## supplementary crystallographic information

### 1. Comment

As a part of our investigation on chemistry of main group elements complexes based on tetradentate ligands (Karlov & Zaitseva, 2001) we obtained and studied the structure of the salen title compound [(salen)AlO-^i^Pr]. The aluminium complex is monomeric (Fig. 1) with a fivefold coordinated Al centre. Its coordination geometry is close to square-based pyramidal with the salen ligand occupying the basal plane and the isopropyloxo group in axial position. It was shown, that the closely related methyloxo compound with an unsubstituted salen ligand [(MeO)(salen)Al]_2_ is dimeric with two bridging µ_2_-alkyloxo ligands (Gurian *et al.* (1991)). In contrast, a similar compound with bulky trimethylphenoxo substituent (2,4,6-Me_3_C_6_H_2_O)(salen)Al exhibited monomeric nature (Atwood *et al.* (1997)). Also all Al compounds with substituted salen ligands, like widely studied complexes of bulky (^t^Bu)_4_salen, are monomeric according to CSD data (Allen, 2002), surely due to the steric hindrances. Thus, it may be assumed that the nuclearity of aluminium complexes with an unsubstituted salen ligand depends on the size of the additional alkyloxo group.

The central metal atom is displaced from the basal N,N,O,O plane by 0.4874 (9) Å. Unexpectedly, the Al-O(3)Alk distance (1.7404 (14) Å) is much shorter than Al-OAr bond lengths from the salen ligand (1.7974 (15) and 1.8094 (14) Å). On the other hand, the Al—O(3)—C(1) angle is quite large with 130.54 (12) °. These values indicate the noticeable degree of π- donation from the apical alkyloxo ligand towards the aluminium centre. The same features were previously found in the structure of the closely related monomeric ethyloxo complex (EtO)(salen(^t^Bu)_4_)Al (Muñoz-Hernandez *et al.* (2000)).

The isopropyloxo group forms an intramolecular hydrogen bond C1—H1A···N1 with H···N with 2.64 Å and C—H···N 116.0 (1)° that is connected with the mutual ecliptic arrangement of N1—Al1 and O3—C1 bonds.

In the crystal packing, the Al-complexes are linked by weak C18—H18A···O3 hydrogen bonds (C···O 3.508 (2) Å, C—H···O 162.9 (1)°). The asymmetric unit contains a solvent dichlormethane molecule that lies on a crystallographic 2-fold axis and forms C4—H4···O1 hydrogen bonds with C···O separations of 3.2539 (18) Å.

### 2. Experimental

The title compound was obtained from reaction of equimolar amounts of Al(O-*i*Pr)_3_ and salen in toluene at reflux as a solid.

^1^H NMR (CDCl_3_): δ 8.24 (s, 2H, N=CH), 7.40–7.33 (m, 2H, aromatic H atoms), 7.16–7.07 (m, 4H, aromatic H atoms), 6.73–6.67 (m, 2H, aromatic H atoms), 4.12–4.02 (m, 2H, NCH_2_), 3.75 (sept, *J* = 6.1 Hz, 1H, OCH), 3.65–3.58 (m, 2H, NCH_2_), 0.91 d (d, *J* = 6.1 Hz, 6H, OCH*Me*_2_) p.p.m..

^13^C NMR (CDCl_3_): δ 168.97 (N=CH), 165.78, 135.46, 132.99, 122.26, 118.74, 116.55 (aromatic carbons), 62.80 (OCH), 54.44 (NCH_2_), 27.33 (OCH*Me*_2_) p.p.m..

### 3. Refinement

All hydrogen atoms were placed in calculated positions and refined using a riding model with C—H = 1.00 Å and *U*_iso_(H) = 1.2*U*_eq_(C) for methyne group; C—H = 0.99 Å and *U*_iso_(H) = 1.2*U*_eq_(C) for methylene groups; C—H = 0.98 Å and *U*_iso_(H) = 1.5*U*_eq_(C) for methyl groups; C—H = 0.95 Å and *U*_iso_(H) = 1.2*U*_eq_(C) for aromatic H atoms.

### Crystal data

**Table:**

[Al(C_16_H_14_N_2_O_2_)(C_3_H_7_O)]·0.5CH_2_Cl_2_	*F*(000) = 3312
*M**_r_* = 394.82	*D*_x_ = 1.378 Mg m^−^^3^
Orthorhombic, *F**d**d*2	Mo *K*α radiation, λ = 0.71073 Å
Hall symbol: F 2 -2d	Cell parameters from 4106 reflections
*a* = 24.427 (3) Å	θ = 2.3–25.6°
*b* = 30.875 (4) Å	µ = 0.27 mm^−^^1^
*c* = 10.0956 (13) Å	*T* = 173 K
*V* = 7614.0 (16) Å^3^	Plate, colourless
*Z* = 16	0.25 × 0.15 × 0.04 mm

### Data collection

**Table:**

Bruker SMART APEXII diffractometer	4069 independent reflections
Radiation source: fine-focus sealed tube	3687 reflections with *I* > 2σ(*I*)
Graphite monochromator	*R*_int_ = 0.032
ω scans	θ_max_ = 27.0°, θ_min_ = 2.3°
Absorption correction: multi-scan (*SADABS*; Bruker, 2008)	*h* = −31→31
*T*_min_ = 0.936, *T*_max_ = 0.989	*k* = −39→39
13794 measured reflections	*l* = −12→12

### Refinement

**Table:**

Refinement on *F*^2^	Secondary atom site location: difference Fourier map
Least-squares matrix: full	Hydrogen site location: inferred from neighbouring sites
*R*[*F*^2^ > 2σ(*F*^2^)] = 0.034	H-atom parameters constrained
*wR*(*F*^2^) = 0.081	*w* = 1/[σ^2^(*F*_o_^2^) + (0.0417*P*)^2^ + 4.145*P*] where *P* = (*F*_o_^2^ + 2*F*_c_^2^)/3
*S* = 1.04	(Δ/σ)_max_ = 0.001
4069 reflections	Δρ_max_ = 0.32 e Å^−^^3^
242 parameters	Δρ_min_ = −0.32 e Å^−^^3^
1 restraint	Absolute structure: Flack (1983), 1872 Friedel pairs
Primary atom site location: structure-invariant direct methods	Absolute structure parameter: −0.09 (7)

### Special details

**Table:**

Geometry. All e.s.d.'s (except the e.s.d. in the dihedral angle between two l.s. planes) are estimated using the full covariance matrix. The cell e.s.d.'s are taken into account individually in the estimation of e.s.d.'s in distances, angles and torsion angles; correlations between e.s.d.'s in cell parameters are only used when they are defined by crystal symmetry. An approximate (isotropic) treatment of cell e.s.d.'s is used for estimating e.s.d.'s involving l.s. planes.
Refinement. Refinement of *F*^2^ against ALL reflections. The weighted *R*-factor *wR* and goodness of fit *S* are based on *F*^2^, conventional *R*-factors *R* are based on *F*, with *F* set to zero for negative *F*^2^. The threshold expression of *F*^2^ > σ(*F*^2^) is used only for calculating *R*-factors(gt) *etc*. and is not relevant to the choice of reflections for refinement. *R*-factors based on *F*^2^ are statistically about twice as large as those based on *F*, and *R*- factors based on ALL data will be even larger.

### Fractional atomic coordinates and isotropic or equivalent isotropic displacement parameters (Å^2^)

**Table:**

	*x*	*y*	*z*	*U*_iso_*/*U*_eq_	
Al1	0.80543 (2)	0.339391 (17)	0.48762 (6)	0.02073 (13)	
O1	0.83307 (5)	0.32093 (4)	0.64267 (15)	0.0274 (3)	
O2	0.74619 (5)	0.30520 (4)	0.51299 (14)	0.0244 (3)	
O3	0.77795 (5)	0.39121 (4)	0.50117 (16)	0.0280 (3)	
N1	0.88207 (6)	0.35385 (5)	0.42972 (17)	0.0233 (4)	
N2	0.80155 (6)	0.32532 (5)	0.29380 (17)	0.0237 (4)	
C1	0.80382 (8)	0.43157 (6)	0.5214 (2)	0.0265 (4)	
H1A	0.8401	0.4311	0.4756	0.032*	
C2	0.76904 (10)	0.46682 (7)	0.4601 (3)	0.0408 (6)	
H2A	0.7647	0.4612	0.3651	0.061*	
H2B	0.7869	0.4949	0.4730	0.061*	
H2C	0.7330	0.4671	0.5025	0.061*	
C3	0.81316 (11)	0.44040 (8)	0.6673 (3)	0.0438 (6)	
H3A	0.8334	0.4162	0.7066	0.066*	
H3B	0.7778	0.4435	0.7120	0.066*	
H3C	0.8343	0.4672	0.6774	0.066*	
C11	0.88005 (8)	0.32767 (6)	0.7048 (2)	0.0247 (4)	
C12	0.88427 (9)	0.31743 (7)	0.8386 (2)	0.0332 (5)	
H12	0.8533	0.3063	0.8842	0.040*	
C13	0.93293 (9)	0.32316 (7)	0.9064 (3)	0.0366 (5)	
H13	0.9349	0.3161	0.9978	0.044*	
C14	0.97909 (9)	0.33922 (7)	0.8422 (3)	0.0350 (5)	
H14	1.0125	0.3426	0.8890	0.042*	
C15	0.97586 (9)	0.35001 (7)	0.7114 (3)	0.0328 (5)	
H15	1.0073	0.3612	0.6678	0.039*	
C16	0.92665 (8)	0.34479 (6)	0.6396 (2)	0.0261 (4)	
C17	0.92542 (8)	0.35630 (6)	0.5013 (2)	0.0270 (4)	
H17	0.9583	0.3662	0.4610	0.032*	
C18	0.88543 (8)	0.36551 (7)	0.2887 (2)	0.0275 (5)	
H18A	0.9239	0.3644	0.2578	0.033*	
H18B	0.8711	0.3951	0.2742	0.033*	
C21	0.70533 (7)	0.29526 (6)	0.4337 (2)	0.0212 (4)	
C22	0.65738 (8)	0.27644 (6)	0.4864 (2)	0.0260 (4)	
H22	0.6560	0.2686	0.5773	0.031*	
C23	0.61261 (8)	0.26946 (6)	0.4067 (2)	0.0304 (5)	
H23	0.5805	0.2571	0.4442	0.036*	
C24	0.61294 (9)	0.27991 (7)	0.2725 (2)	0.0356 (5)	
H24	0.5809	0.2767	0.2200	0.043*	
C25	0.66070 (9)	0.29500 (7)	0.2186 (2)	0.0346 (5)	
H25	0.6622	0.3008	0.1262	0.041*	
C26	0.70752 (8)	0.30217 (6)	0.2965 (2)	0.0270 (4)	
C27	0.75757 (8)	0.31427 (6)	0.2327 (2)	0.0269 (4)	
H27	0.7583	0.3141	0.1387	0.032*	
C28	0.85114 (8)	0.33286 (7)	0.2152 (2)	0.0293 (4)	
H28A	0.8413	0.3441	0.1264	0.035*	
H28B	0.8717	0.3055	0.2038	0.035*	
Cl1	0.96134 (3)	0.46418 (3)	0.61765 (8)	0.0636 (2)	
C4	1.0000	0.5000	0.5209 (3)	0.0304 (7)	
H4	0.9758	0.5163	0.4650	0.036*	

### Atomic displacement parameters (Å^2^)

**Table:**

	*U*^11^	*U*^22^	*U*^33^	*U*^12^	*U*^13^	*U*^23^
Al1	0.0173 (2)	0.0212 (3)	0.0238 (3)	−0.0013 (2)	0.0013 (2)	0.0001 (2)
O1	0.0219 (7)	0.0353 (8)	0.0249 (8)	−0.0063 (6)	−0.0031 (6)	0.0037 (6)
O2	0.0216 (6)	0.0264 (6)	0.0252 (8)	−0.0046 (5)	−0.0003 (6)	0.0029 (6)
O3	0.0210 (6)	0.0211 (6)	0.0418 (9)	−0.0013 (5)	0.0022 (7)	−0.0033 (7)
N1	0.0209 (8)	0.0223 (8)	0.0266 (9)	−0.0007 (6)	0.0031 (7)	0.0016 (7)
N2	0.0232 (8)	0.0241 (8)	0.0238 (9)	−0.0005 (6)	0.0037 (7)	0.0019 (7)
C1	0.0222 (9)	0.0229 (9)	0.0344 (12)	−0.0023 (7)	0.0027 (9)	−0.0023 (8)
C2	0.0459 (13)	0.0263 (10)	0.0502 (17)	0.0013 (9)	−0.0088 (12)	−0.0008 (10)
C3	0.0522 (15)	0.0374 (12)	0.0416 (15)	−0.0125 (11)	−0.0065 (12)	−0.0009 (11)
C11	0.0232 (9)	0.0233 (9)	0.0276 (12)	0.0007 (8)	−0.0031 (8)	−0.0012 (8)
C12	0.0300 (11)	0.0373 (12)	0.0321 (13)	−0.0002 (9)	−0.0016 (9)	0.0001 (10)
C13	0.0365 (12)	0.0431 (13)	0.0301 (13)	0.0050 (10)	−0.0075 (10)	−0.0040 (10)
C14	0.0272 (11)	0.0368 (12)	0.0409 (14)	0.0030 (9)	−0.0107 (10)	−0.0084 (10)
C15	0.0227 (9)	0.0293 (10)	0.0464 (15)	−0.0009 (8)	−0.0030 (9)	−0.0045 (10)
C16	0.0225 (9)	0.0226 (9)	0.0332 (12)	0.0004 (7)	−0.0024 (9)	−0.0028 (8)
C17	0.0200 (9)	0.0221 (9)	0.0390 (13)	−0.0003 (7)	0.0039 (9)	0.0003 (9)
C18	0.0214 (10)	0.0280 (10)	0.0330 (12)	−0.0003 (8)	0.0063 (9)	0.0068 (9)
C21	0.0205 (9)	0.0157 (8)	0.0275 (10)	0.0022 (7)	−0.0015 (8)	−0.0005 (7)
C22	0.0271 (10)	0.0207 (9)	0.0302 (11)	−0.0002 (7)	0.0022 (10)	−0.0006 (9)
C23	0.0222 (10)	0.0257 (10)	0.0433 (14)	−0.0029 (8)	0.0033 (10)	−0.0030 (9)
C24	0.0243 (10)	0.0413 (12)	0.0411 (14)	−0.0034 (9)	−0.0088 (10)	−0.0027 (10)
C25	0.0309 (11)	0.0429 (12)	0.0299 (12)	−0.0047 (9)	−0.0059 (10)	0.0020 (10)
C26	0.0268 (10)	0.0268 (10)	0.0273 (12)	−0.0015 (8)	−0.0016 (9)	−0.0002 (8)
C27	0.0280 (10)	0.0300 (10)	0.0228 (11)	−0.0008 (8)	0.0009 (9)	0.0001 (9)
C28	0.0272 (10)	0.0327 (10)	0.0280 (12)	0.0020 (8)	0.0064 (9)	0.0044 (9)
Cl1	0.0662 (4)	0.0686 (4)	0.0559 (5)	−0.0116 (4)	0.0136 (4)	0.0262 (4)
C4	0.0347 (16)	0.0328 (15)	0.0236 (16)	−0.0003 (12)	0.000	0.000

### Geometric parameters (Å, º)

**Table:**

Al1—O3	1.7404 (14)	C14—C15	1.363 (4)
Al1—O1	1.7974 (15)	C14—H14	0.9500
Al1—O2	1.8094 (14)	C15—C16	1.413 (3)
Al1—N2	2.0066 (18)	C15—H15	0.9500
Al1—N1	2.0115 (17)	C16—C17	1.442 (3)
O1—C11	1.324 (2)	C17—H17	0.9500
O2—C21	1.315 (2)	C18—C28	1.506 (3)
O3—C1	1.412 (2)	C18—H18A	0.9900
N1—C17	1.284 (3)	C18—H18B	0.9900
N1—C18	1.471 (3)	C21—C26	1.403 (3)
N2—C27	1.285 (3)	C21—C22	1.412 (3)
N2—C28	1.467 (2)	C22—C23	1.375 (3)
C1—C2	1.513 (3)	C22—H22	0.9500
C1—C3	1.515 (3)	C23—C24	1.392 (3)
C1—H1A	1.0000	C23—H23	0.9500
C2—H2A	0.9800	C24—C25	1.369 (3)
C2—H2B	0.9800	C24—H24	0.9500
C2—H2C	0.9800	C25—C26	1.406 (3)
C3—H3A	0.9800	C25—H25	0.9500
C3—H3B	0.9800	C26—C27	1.431 (3)
C3—H3C	0.9800	C27—H27	0.9500
C11—C12	1.392 (3)	C28—H28A	0.9900
C11—C16	1.417 (3)	C28—H28B	0.9900
C12—C13	1.383 (3)	Cl1—C4	1.7521 (19)
C12—H12	0.9500	C4—Cl1^i^	1.7520 (19)
C13—C14	1.392 (3)	C4—H4	0.9600
C13—H13	0.9500		
			
O3—Al1—O1	111.59 (8)	C15—C14—H14	120.3
O3—Al1—O2	102.52 (7)	C13—C14—H14	120.3
O1—Al1—O2	89.55 (7)	C14—C15—C16	121.2 (2)
O3—Al1—N2	104.92 (8)	C14—C15—H15	119.4
O1—Al1—N2	142.97 (7)	C16—C15—H15	119.4
O2—Al1—N2	88.51 (7)	C15—C16—C11	119.2 (2)
O3—Al1—N1	100.24 (7)	C15—C16—C17	119.13 (19)
O1—Al1—N1	88.50 (7)	C11—C16—C17	121.66 (18)
O2—Al1—N1	156.20 (7)	N1—C17—C16	123.21 (18)
N2—Al1—N1	78.96 (7)	N1—C17—H17	118.4
C11—O1—Al1	133.47 (13)	C16—C17—H17	118.4
C21—O2—Al1	131.06 (13)	N1—C18—C28	106.40 (16)
C1—O3—Al1	130.54 (12)	N1—C18—H18A	110.4
C17—N1—C18	118.95 (17)	C28—C18—H18A	110.4
C17—N1—Al1	128.11 (15)	N1—C18—H18B	110.4
C18—N1—Al1	112.80 (13)	C28—C18—H18B	110.4
C27—N2—C28	118.22 (18)	H18A—C18—H18B	108.6
C27—N2—Al1	124.39 (15)	O2—C21—C26	122.43 (17)
C28—N2—Al1	117.03 (13)	O2—C21—C22	119.78 (18)
O3—C1—C2	108.93 (17)	C26—C21—C22	117.78 (18)
O3—C1—C3	111.52 (18)	C23—C22—C21	120.3 (2)
C2—C1—C3	110.63 (19)	C23—C22—H22	119.9
O3—C1—H1A	108.6	C21—C22—H22	119.9
C2—C1—H1A	108.6	C22—C23—C24	121.9 (2)
C3—C1—H1A	108.6	C22—C23—H23	119.0
C1—C2—H2A	109.5	C24—C23—H23	119.0
C1—C2—H2B	109.5	C25—C24—C23	118.1 (2)
H2A—C2—H2B	109.5	C25—C24—H24	121.0
C1—C2—H2C	109.5	C23—C24—H24	121.0
H2A—C2—H2C	109.5	C24—C25—C26	121.6 (2)
H2B—C2—H2C	109.5	C24—C25—H25	119.2
C1—C3—H3A	109.5	C26—C25—H25	119.2
C1—C3—H3B	109.5	C21—C26—C25	119.85 (19)
H3A—C3—H3B	109.5	C21—C26—C27	121.08 (19)
C1—C3—H3C	109.5	C25—C26—C27	119.0 (2)
H3A—C3—H3C	109.5	N2—C27—C26	124.6 (2)
H3B—C3—H3C	109.5	N2—C27—H27	117.7
O1—C11—C12	119.22 (19)	C26—C27—H27	117.7
O1—C11—C16	122.36 (19)	N2—C28—C18	107.38 (17)
C12—C11—C16	118.41 (19)	N2—C28—H28A	110.2
C13—C12—C11	121.0 (2)	C18—C28—H28A	110.2
C13—C12—H12	119.5	N2—C28—H28B	110.2
C11—C12—H12	119.5	C18—C28—H28B	110.2
C12—C13—C14	120.8 (2)	H28A—C28—H28B	108.5
C12—C13—H13	119.6	Cl1^i^—C4—Cl1	112.20 (18)
C14—C13—H13	119.6	Cl1^i^—C4—H4	109.3
C15—C14—C13	119.4 (2)	Cl1—C4—H4	109.0

Symmetry code: (i) −*x*+2, −*y*+1, *z*.

### Hydrogen-bond geometry (Å, º)

**Table:**

*D*—H···*A*	*D*—H	H···*A*	*D*···*A*	*D*—H···*A*
C1—H1*A*···N1	1.00	2.64	3.204 (3)	116
C18—H18*A*···O3^ii^	0.99	2.55	3.508 (2)	163
C4—H4···O1^iii^	0.96	2.33	3.2539 (18)	160

Symmetry codes: (ii) *x*+1/4, −*y*+3/4, *z*−1/4; (iii) −*x*+7/4, *y*+1/4, *z*−1/4.
